# The Role of IL-17 in the Association between Pneumococcal Pneumonia and Allergic Sensitization

**DOI:** 10.1155/2011/709509

**Published:** 2011-11-17

**Authors:** Hongxia Zhao, Cheol-In Kang, Mark S. Rouse, Robin Patel, Hirohito Kita, Young J. Juhn

**Affiliations:** ^1^Division of Community Pediatric and Adolescent Medicine, Department of Pediatric and Adolescent Medicine, Mayo Clinic, Rochester, MN, USA; ^2^Department of Pediatrics, Shenzhen Maternal and Child Health Care Hospital Affiliated to Nanfang Medical University, Shenzhen 518028, China; ^3^Division of Infectious Diseases, Samsung Medical Center, Sungkyunkwan University School of Medicine, Seoul 135-710, Republic of Korea; ^4^Division of Infectious Diseases, Department of Medicine, Mayo Clinic, Rochester, MN, USA; ^5^Division of Clinical Microbiology, Department of Laboratory Medicine and Pathology, Mayo Clinic, Rochester, MN, USA; ^6^Division of Allergic Diseases, Department of Medicine, Mayo Clinic, Rochester, MN, USA; ^7^Department of Pediatric and Adolescent Medicine, College of Medicine, Mayo Clinic, 200 First Street SW, Rochester, MN 55905, USA

## Abstract

Interleukin- (IL-) 17 is important in the development of asthma and host defense against pneumococci. We determined the role of IL-17 in the risk of pneumococcal pneumonia. We challenged mice intranasally with a bioluminescent *Streptococcus pneumoniae* strain after sensitization and challenge with ovalbumin (OVA). We measured the levels of cytokines, including IL-17 (pg/mL), in the lung homogenate in experimental mice with and without OVA sensitization/challenge, as well as those with and without pneumococcal pneumonia. IL-17 levels were significantly lower in OVA-sensitized/challenged mice (9.69 ± 1.49), compared to the control mice (20.92 ± 1.82, *P* < 0.001). In our overall analysis, including IL-4 and IL-17 levels and OVA sensitization/challenge, IL-4 levels (OR: 81.9, 95%CI: 4.3–1523 per increment of 1.0 pg/mL, *P* = 0.003) were more significant than IL-17 levels (OR: 1.1, 95%CI: 1.03–1.17 per increment of 1.0 pg/mL, *P* = 0.003) in determining the risk of pneumococcal pneumonia. IL-17 levels result in a much smaller impact on the risk for pneumococcal pneumonia, compared to IL-4 levels.

## 1. Introduction

*Streptococcus pneumoniae* is a leading cause of bacterial pneumonia, meningitis, and sepsis and has caused substantial morbidity and mortality in the United States and worldwide, especially in young children. Recent estimates of childhood deaths caused by *Streptococcus pneumoniae* range from 70,000 to 1 million every year worldwide, and *S. pneumoniae* caused around 11% of all deaths in children aged 1–59 months [1]. In 2000, about 14.5 million episodes of serious pneumococcal disease were estimated to occur, and pneumococcal disease caused about 826,000 deaths in children aged 1–59 months [1]. In the US, 40,000 cases of fatal pneumococcal infections per year have been reported [2]. Recently some studies reported that individuals with asthma have a significantly increased risk of invasive pneumococcal disease, compared to those without asthma [3, 4]. Thus, the Advisory Committee on Immunization Practices has a recommendation for the use of 23-valent pneumococcal polysaccharide vaccine (PPV23) for the prevention of invasive pneumococcal disease in asthmatics [5]. At present, little is known about the mechanisms underlying the increased risk of invasive pneumococcal disease among individuals with asthma. We previously reported that a T-helper 2 (Th2) predominant immune response (e.g., IL-4) to OVA sensitization was a significant risk factor for pneumococcal infection, whereas allergic sensitization itself was protective against pneumococcal pneumonia [6]. Others also reported that IL-4 was associated with diminished host defense against *Pseudomonas* lung infection in mice [7]. Therefore, while Th2 cytokines such as IL-4 are associated with a risk of bacterial pneumonia in mice, how allergic sensitization provides a protective effect against pneumococcal pneumonia in mice is not well understood. One potential hypothesis is that allergic sensitization-induced IL-17 may provide a protective effect against pneumococcal disease because IL-17 is a cytokine that enhances host defense against pneumococcal infection. T-helper 17 (Th17) cells are a newly discovered CD4^+^ helper T-cell subset that produce IL-17 and are involved in host defense against bacterial infection, mainly through the recruitment and activation of neutrophils [8]. IL-17 is an important inflammatory mediator in the development of asthma [9, 10] and protection from pneumococcal colonization [11, 12]. Indeed, recent studies report that allergic sensitization with OVA in mice induced secretion of IL-17 from macrophages and elevation of IL-17 levels in BAL fluid [10, 13], but production of IL-17 by Th17 cells (particularly IL-23-dependent IL-17 secretion) was negatively regulated by IL-4 [14]. Therefore, we hypothesize that elevated IL-17 levels in BAL induced by allergic sensitization may provide a protective effect on the development of pneumococcal pneumonia. To test this hypothesis, we investigated the role of IL-17 in both airway inflammation and pneumococcal pneumonia using a murine model.

## 2. Materials and Methods

### 2.1. Experiment

Experimental details have been previously reported [6] the study was approved by the Mayo Clinic IACUC. Briefly, pathogen-free 6–8-week-old female BALB/c mice were sensitized by intraperitoneal (i.p.) injection of 20 *μ*g OVA adsorbed to 1 mg aluminum hydroxide gel (in a volume of 100 *μ*L) on days 0 and 7. Then, experimental mice were intranasally challenged with 100 *μ*g OVA in 50 *μ*L phosphate-buffered saline (PBS) under light tribromoethanol anesthesia on days 15, 16, and 17. Control mice received i.p. injection of PBS with aluminum hydroxide gel but no intranasal challenge. Three days after OVA challenge, the OVA-sensitized/challenged mice and control mice were challenged with *S. pneumoniae* A 66.1 serotype 3 (1.5 × 104 to 3 × 104 cfu), made bioluminescent by integration of a modified lux operon into its chromosome (Xen 10, Caliper Life Sciences, Hopkinton, Mass, USA) [15]. Pneumococcal pneumonia was defined as the presence of bioluminescence and positive pneumococcal culture from normally sterile body tissues (spleen or brain). Cytokine and chemokine levels in the lung tissues were determined by Bio-Plex or ELISA assays.

### 2.2. Outcome Measures and Statistical Analysis

To examine the impact of OVA sensitization/challenge on IL-17 levels in the lung, we compared IL-17 levels between OVA-sensitized/challenged and control mice after stratification into mice that developed pneumococcal pneumonia and those that did not. To assess the role of IL17 in the risk of pneumococcal disease, we compared IL-17 levels between mice with and without pneumococcal pneumonia and determined the correlation between IL-17 levels and colony counts of pneumococci from lung and spleen (log_10_ CFU) using Pearson's correlation coefficient. To determine the relationship between IL-4 and IL-17, we calculated Pearson's correlation coefficient between IL-17 and Th2 cytokines.

To examine whether the impact of IL-17 on pneumococcal infection is independent of sensitization and challenge with OVA and IL-4, data were fitted to a logistic regression. We calculated odds ratios (ORs) and corresponding 95% confidence intervals for OVA sensitization/challenge, IL-17 levels, IL-4 levels, and the interaction term between OVA sensitization/challenge and IL-4 to identify factors associated with the risk of pneumococcal pneumonia. All statistical significance was tested at a two-tailed alpha error of  0.05.

## 3. Results

### 3.1. Relationship between IL-17 Levels in Lung, OVA Sensitization/Challenge, and Pneumococcal Pneumonia

IL-17 levels were significantly lower in OVA-sensitized/challenged mice, compared to the control mice (9.69 ± 1.49 versus 20.92 ± 1.82 pg/mL, *P* < 0.001) and were elevated in mice with compared to those without pneumococcal pneumonia (20.53 ± 1.96 versus 10.28 ± 1.44 pg/mL, *P* < 0.001).

The results stratified by pneumococcal pneumonia status are summarized in Table 1. IL-17 level was lower in OVA-sensitized/challenged mice, compared to control mice in the mice without pneumococcal pneumonia (*P* = 0.001). However, there was no difference in IL-17 levels between OVA-sensitized/challenged mice and control mice in mice with pneumococcal pneumonia (*P* = 0.13). In OVA-sensitized/challenged mice, the IL-17 level was higher in mice that developed pneumococcal pneumonia than those that did not (*P* = 0.01). However, in the control mice, the IL-17 level was not associated with pneumococcal pneumonia (*P* = 0.21).

**Figure 1:**
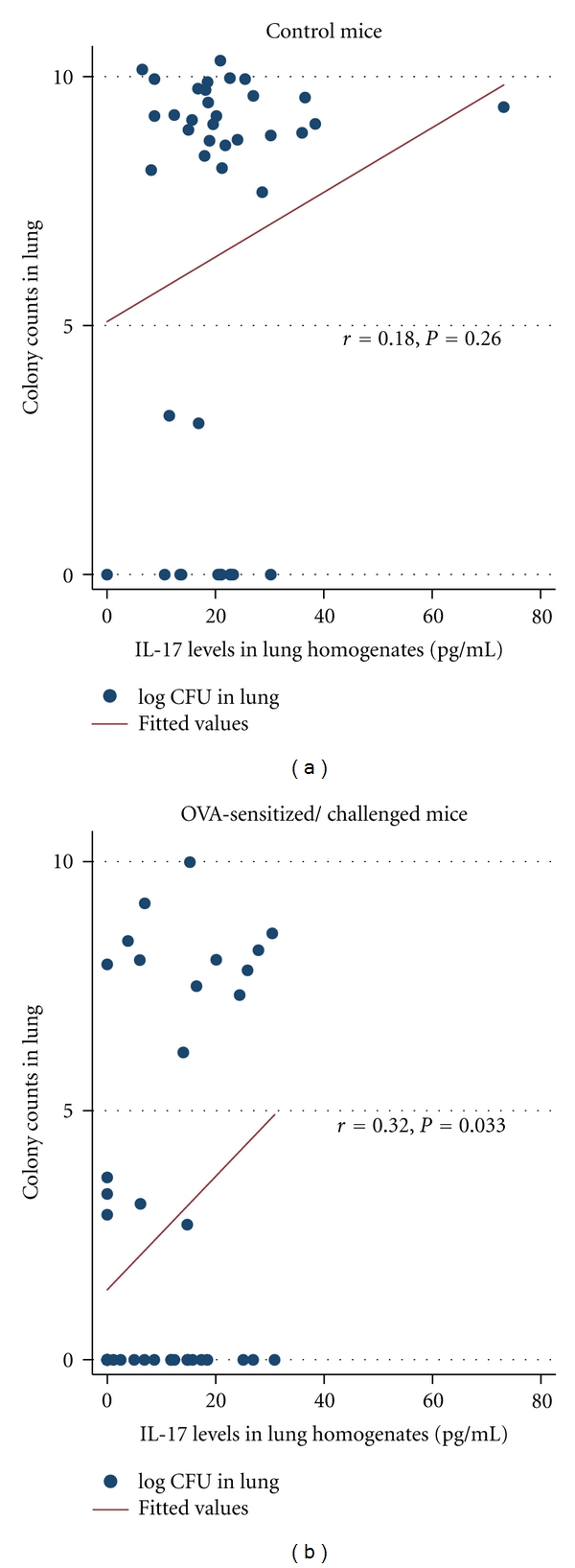
Correlation between IL17 levels in lung homogenates and pneumococcal colony counts in lung. To assess the effect of IL-17 on the risk of pneumococcal pneumonia, the correlation between IL-17 levels in the lung and pneumococcal colony count in the lung was examined. When mice developed pneumococcal pneumonia by bioluminescence or expired, we measured IL-17 levels and pneumococcal colony counts in the lung. As shown in the figure, IL-17 levels in the lung were positively correlated with pneumococcal colony counts in lungs of mice with OVA sensitization/challenge (*r* = 0.32, *P* = 0.033), but not the controls. OVA sensitization/challenge modified the effect of IL-17 on the risk of pneumococcal pneumonia. log CFU; log_10_ colony forming units of pneumococci.

**Figure 2:**
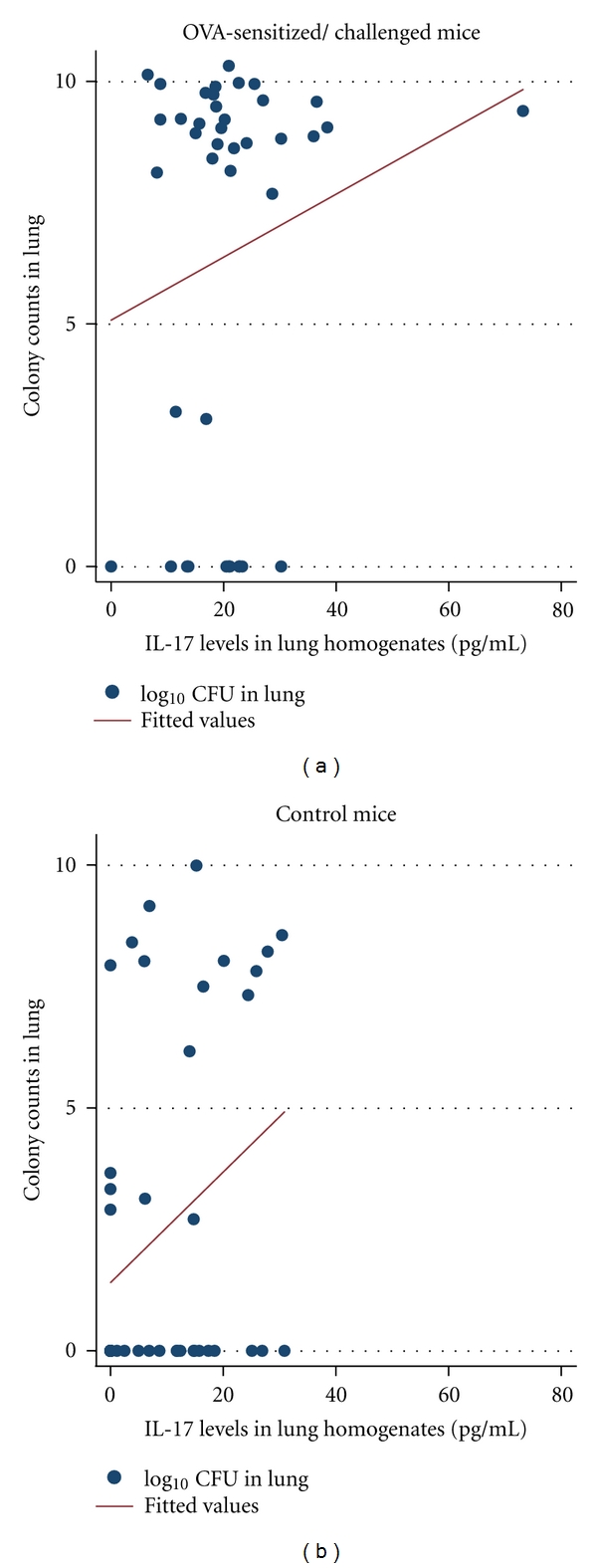
Correlation between IL17 levels in lung homogenates and pneumococcal colony counts in spleen. To assess the effect of IL-17 on the risk of invasive pneumococcal disease instead of pneumococcal pneumonia, the correlation between IL-17 levels in the lung and pneumococcal colony count in the spleen, which should be sterile normally, was assessed. When mice developed invasive pneumococcal disease involving the spleen (by bioluminescence) or expired, we measured IL-17 levels and pneumococcal colony counts in the lung. There was a significant correlation between IL-17 levels in the lung homogenate and pneumococcal colony counts in the spleen among the OVA-sensitized/challenged mice (*r* = 0.36, *P* = 0.014), but no significant correlation was found in the control mice. OVA sensitization/challenge modified the effect of IL-17 on the risk of invasive pneumococcal disease. *log CFU: lo4g_10_ colony forming units of pneumococci.

IL-17 levels were positively correlated with pneumococcal colony counts in the lung and spleen in the mice with OVA sensitization/challenge, but not the control mice (Figures 1 and 2).

### 3.2. Correlation between IL-17 Levels and Other Cytokines and Chemokines in Lung

IL-17 levels were positively correlated with IL-1*α* (*r* = 0.31), IL-13 (0.32), and RANTES (*r* = 0.37) levels in the lungs of sensitized mice (*P* < 0.05), whereas IL-17 levels were inversely correlated with IL-4 levels in the lungs of mice without pneumococcal pneumonia, a finding which approached statistical significance (*r* = −0.29, *P* = 0.05). There was no correlation between IL-17 and other cytokines (IL-1, IL-2, IL-5, IL-6, IL-10, IFN-*γ*, and TNF-*α*) or chemokines (GM-CSF, EOTAXIN, and MIP-1) in lung homogenates.

### 3.3. Factors Associated with the Risk of Pneumococcal Pneumonia

The results on the influence of IL-17, IL-4, and OVA sensitization/challenge on the risk of pneumococcal pneumonia are summarized in Table 2. The mean level (± standard error) of IL-4 in lung homogenates among mice with pneumococcal pneumonia was 1.09 ± 0.14 pg/mL whereas that among mice without pneumococcal pneumonia was 0.67 ± 0.06 pg/mL (*P* = 0.0037). Therefore, adjusting for the impact of IL-4 and OVA sensitization/challenge on the risk on pneumococcal pneumonia, IL-17 minimally influenced the risk of pneumococcal pneumonia. OVA sensitization/challenge was not significantly associated with the risk of pneumococcal pneumonia, but, as we previously reported, IL-4 was still significantly associated with the risk of pneumococcal pneumonia adjusting for IL-17 and OVA sensitization/challenge.

## 4. Discussion

In our study, sensitization/challenge with OVA reduced the risk of pneumococcal pneumonia (28% versus 57%) and the number of pneumococci in the lung homogenates of mice with pneumococcal pneumonia, compared to control mice. This reduced risk of pneumococcal pneumonia in OVA-sensitized/challenged mice was minimally accounted for by IL-17 levels in lung, but IL-4 levels in lung were more significantly associated with the risk of pneumococcal pneumonia than IL-17.

Our results suggest that IL-17 levels were lower in OVA-sensitized/challenged mice than the control mice, particularly in mice without pneumococcal pneumonia. However, it is uncertain whether OVA sensitization/challenge failed to induce IL-17 since we did not have a baseline measure of IL-17. However, Th2 cytokines showed an inconsistent relationship with IL-17 levels. There was no correlation between IL-5 and IL-17 levels. IL-4 was inversely correlated with IL-17 levels whereas IL-13 was positively correlated with IL-17 levels in the lung. Therefore, in our mouse model, allergic sensitization might not significantly increase IL-17 levels in the lung homogenate but the inconsistent effects of Th2 cytokines on IL-17 levels. Previous studies reported that OVA sensitization/challenge increases IL-17 levels in lung [9, 10]. However, Schnyder-Candrian et al. reported that IL-17 has a dual role: it is essential during antigen sensitization to establish allergic asthma, but, in sensitized mice, IL-17 attenuates the allergic response by inhibiting chemokines [10]. In our study, IL-17 levels in the lung were positively correlated with IL-1 levels but inversely correlated with IL-4 levels, findings which are consistent with the literature [14, 16–18].

For the relationship between IL-17 levels in the lung and the risk of pneumococcal pneumonia, in our study, IL-17 levels in the lung were associated with an increased risk of pneumococcal pneumonia, particularly in OVA-sensitized/challenged mice. IL-17 levels in the lung are positively correlated with pneumococcal colony counts from lung and spleen as summarized in Figures 1 and 2, and there was a positive association between IL-17 levels and pneumococcal pneumonia as summarized in Tables 1 and 2. In interpreting these results, IL-17 levels in the lung might reflect host response to pneumococcal pneumonia (i.e., IL-17 levels induced by pneumococcal pneumonia) instead of causing an increased risk of pneumococcal pneumonia. However, we observed increased IL-17 levels in mice with pneumococcal pneumonia only among the group of OVA-sensitized/challenged mice, but IL-17 levels did not differ between mice with and without pneumococcal pneumonia in the control mice as shown in Table 1. Also, IL-17 levels were not correlated with pneumococcal colony counts in lung and spleen in the control mice. Therefore, IL-17 levels in the lung might not solely reflect the host response to pneumococcal pneumonia. The role of IL-17 in host defense from microbial infections including pneumococcal infection has been well supported by the literature [11, 12, 19, 20]. IL-17 participates in host defense through regulation of innate immunity (e.g., recruitment of neutrophils and induction of antimicrobial peptides) and cell-mediated immunity (e.g., T-cell activation) [8, 20]. However, our study results were in contrast with these studies and suggest a potential adverse effect of IL-17 on host defense. Our study is limited in addressing this unexpected finding, and we can only speculate as to the potential explanation. As an organism-specific mechanism, a recent study reported that IL-17 up-regulates expression of polymeric immunoglobulin receptor (PIgR) by epithelial cells, a receptor for pneumococcal cholin-binding protein A (CbpA) [21]. CbpA has been known as an important pneumococcal adhesin to certain receptors (e.g., PIgR as well as platelet-activating factor receptor) to invade through reverse epithelial transcytosis [22, 23]. Thus, upregulation of PIgR may increase binding of pneumococci to the epithelial cell surface and allow pneumococci to invade epithelial cells and inner tissues, resulting in serious infections [24]. Also, Th2 cytokines such as IL-4 upregulate PIgR and may further enhance invasion of pneumococci in the presence of IL-17 which may account for the positive correlation between IL-17 and pneumococcal colony count in the lung and spleen only among OVA-sensitized/challenged but not control mice, as observed in our study [22]. As a nonspecific mechanism for the negative influence of IL-17 on the risk of pneumococcal pneumonia, IL-17 has been reported to promote inflammation by inducing various proinflammatory cytokines and chemokines, recruiting neutrophils, enhancing antibody production, and activating T cells [24]. The etiologic involvement of IL-17 in certain autoimmune conditions such as rheumatoid arthritis [25] and multiple sclerosis [26] has been suggested. Therefore, IL-17 might result in significant airway inflammation damaging lung architecture and making the host susceptible to microbial infections. Indeed, previous studies showed that IL-17 was associated with increased risks of *Aspergillus fumigatus* infection and diminished antifungal immunity [27]. However, in our study, the overall impact of IL-17 on the risk of pneumococcal pneumonia was minimal (OR: 1.10), taking into account the role of IL-4 and OVA sensitization/challenge as summarized in Table 2.

As we previously reported, IL-4 levels in the lung were still a significant factor for the risk of pneumococcal pneumonia in our mouse model, adjusting for the role of IL-17 and OVA sensitization/challenge. The adverse effect of IL-4 on the risk of microbial infections has been well supported by the literature [28, 29]. Thus, as discussed above, given the dual effects (protective and provocative) of IL-17 on the risk of pneumococcal pneumonia, IL-4 (and attenuated production of proinflammatory cytokines by reciprocal inhibition by Th2 cytokines) may play a more important role in determining the risk of pneumococcal pneumonia than IL-17.

The main strengths of our study were a large sample size of mice ensuring sufficient statistical power and use of a luminescence technique allowing us to identify pneumococcal pneumonia without sacrificing mice. Our study also had limitations. There was no measurement for IL-17 levels in the lung at baseline (time zero), and IL-17 levels were measured at different time points depending on disease progress of mice, which may influence the interpretation of the results. Another limitation of the animal model of this study was acute airway inflammation status, and the result might not be applicable for asthma in human, a chronic airway inflammation.

In conclusion, our study results highlight that allergic sensitization may not induce IL-17, and IL-17 levels in the lung may result in a negative influence on host defense against pneumococcal pneumonia, albeit minimal. However, IL-4 levels in the lung are still more important for determining the risk of pneumococcal pneumonia than IL-17 and allergic sensitization. Given the limitations of our study, further studies are needed to clarify the role of IL-17 in the risk of pneumococcal disease.

## Figures and Tables

**Table 1:** Comparison of IL-17 among the OVA-sensitized/challenged mice and the control mice stratified by pneumococcal pneumonia status.

	OVA-sensitized/challenged mice	Control mice	*P* value
Mice with pneumococcal pneumonia	15.94 ± 2.92	22.5 ± 2.44	0.13
Mice without pneumococcal pneumonia	7.43 ± 1.59	17.52 ± 2.12	0.001
*P* value	0.01	0.21	

Data are presented as mean ± standard error (pg/mL). The lung tissues of mice at the time of death or pneumococcal pneumonia or those of mice without pneumococcal pneumonia at day 7 were homogenized with 2 mL of PBS and frozen at −20°C for subsequent cytokine analysis. IL-17 levels in lung homogenates were determined by ELISA methods.

**Table 2:** Logistic regression models assessing the impact of each variable on the risk of pneumococcal pneumonia.

Variables	Odds ratios (95%CI)*	*P* value
IL-17 (per an increment of 1.0 pg/mL)	1.10 (1.03–1.17)	0.003
OVA sensitization/challenge	4.03 (0.46–35.07)	0.21
IL-4 (per an increment of 1.0 pg/mL)	81.9 (4.30–1523.87)	0.003

The logistic regression model was adjusted for the interaction term between IL-4 and OVA sensitization/challenge.

## Abbreviations

OR: Odds ratio
95%CI: 95% confidence interval
CFU: Colony forming unit
OVA: Ovalbumin.
